# Direct interaction in digital interactive media and stock performance: Evidence from Panorama

**DOI:** 10.1371/journal.pone.0302448

**Published:** 2024-05-28

**Authors:** Jinshui Huang, Jun Wang, Xiaoman Jin

**Affiliations:** 1 School of Management Science and Engineering, Southwestern University of Finance and Economics, Chengdu, Sichuan, China; 2 School of Finance, Hebei University of Economics and Business, Shijiazhuang, Hebei, China; Roma Tre University: Universita degli Studi Roma Tre, ITALY

## Abstract

Media information plays an essential role in the stock market. Recent financial research has verified that media information could shock stock price by influencing investors’ expectation. Now, a new type of interactive media, called Digital Interactive Media (DIM), is popular in Chinese stock market and becomes the main channel for investors to understand listed companies. Unlike general news media or investor forums, DIM enables direct interaction between listed companies and investors. In the modern society where digital economy is booming, media information would largely affect investors’ decisions. Therefore, it is urgent to use natural language processing (NLP) technology to deconstruct the massive questions and answers (Q&A) interactive information in DIM and extract valuable factors that affect stock prices and stock performances to explore the influence mechanism of digital interactive information on stock performances. This paper firstly uses web crawling technology to obtain approximately 110000 Q&A text information from the digital interactive platform (‘Panoramic Network’) from 2015 to 2021. Then we use big data text analysis technology and emotional quantification technology to extract valuable influencing factors from the massive text. A Multiple Linear Regression (MLR) model was created to explore specific influence mechanism of digital interactive information on stock price performance. The empirical results show that the emotions implicit in investors’ questions do not significantly impact stock performance. However, the emotions and attitudes of the answers by listed companies can significantly affect corresponding stock prices, which indirectly confirms the Proximate Cause Effect of behavioral finance. This effect is particularly evident in the stock prices on the current trading day and the next trading day. In the Robustness Test, this paper replaces dependent variable and adds relevant control variables, and the conclusion remains valid. In the Endogeneity Test, this paper selects sample data before the launch of Panorama Network in 2014 as a comparison, and uses a Difference-in-Difference (DID) model to prove the significant impact of the launch of Panorama Network on Chinese stock market. In the Heterogeneity Test, the paper classifies the market value, region, and industry of listed companies and regressed the sub samples, once again confirming the reliability of the empirical conclusions. The results of Robustness Test, Endogeneity Test, and Heterogeneity Test conducted in this paper all support empirical conclusions.

## Introduction

**Fig 1:**
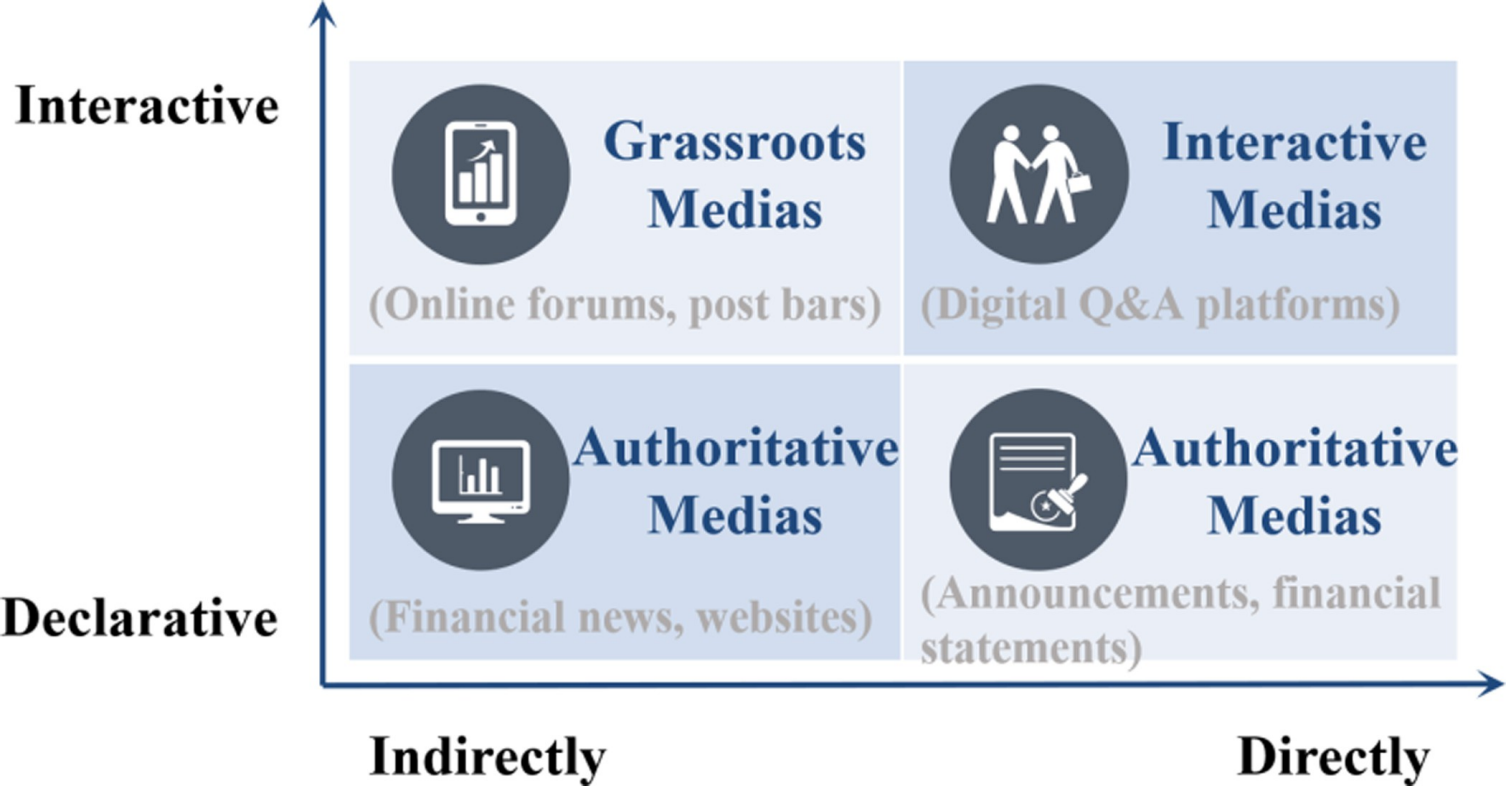
Coordinate chart of media information characteristics at different media development stages.

Behavioral finance research has verified that media information, as an form of external information, continuously shocks stock market and listed companies [1]. With the unceasing upgrading of digital economy, the mainstream media platforms that affect stock market are also evolving [2, 3]. It has mainly experienced the following stages of development in China: firstly, official financial website that releases "authoritative" news to investors unilaterally, then social media that investors can freely release and discuss information, and then evolved into the latest digital interactive media that listed companies and investors can directly interact with, which has become the most important information release channel for listed companies in China. The different information characteristics of medias at different stages of media development are shown in Fig 1. In a series of digital interactive medias, "Panorama", "Hudong-Yi", "E-Hudong", and "Asking DongMi" are the representatives.

**Fig 2:**
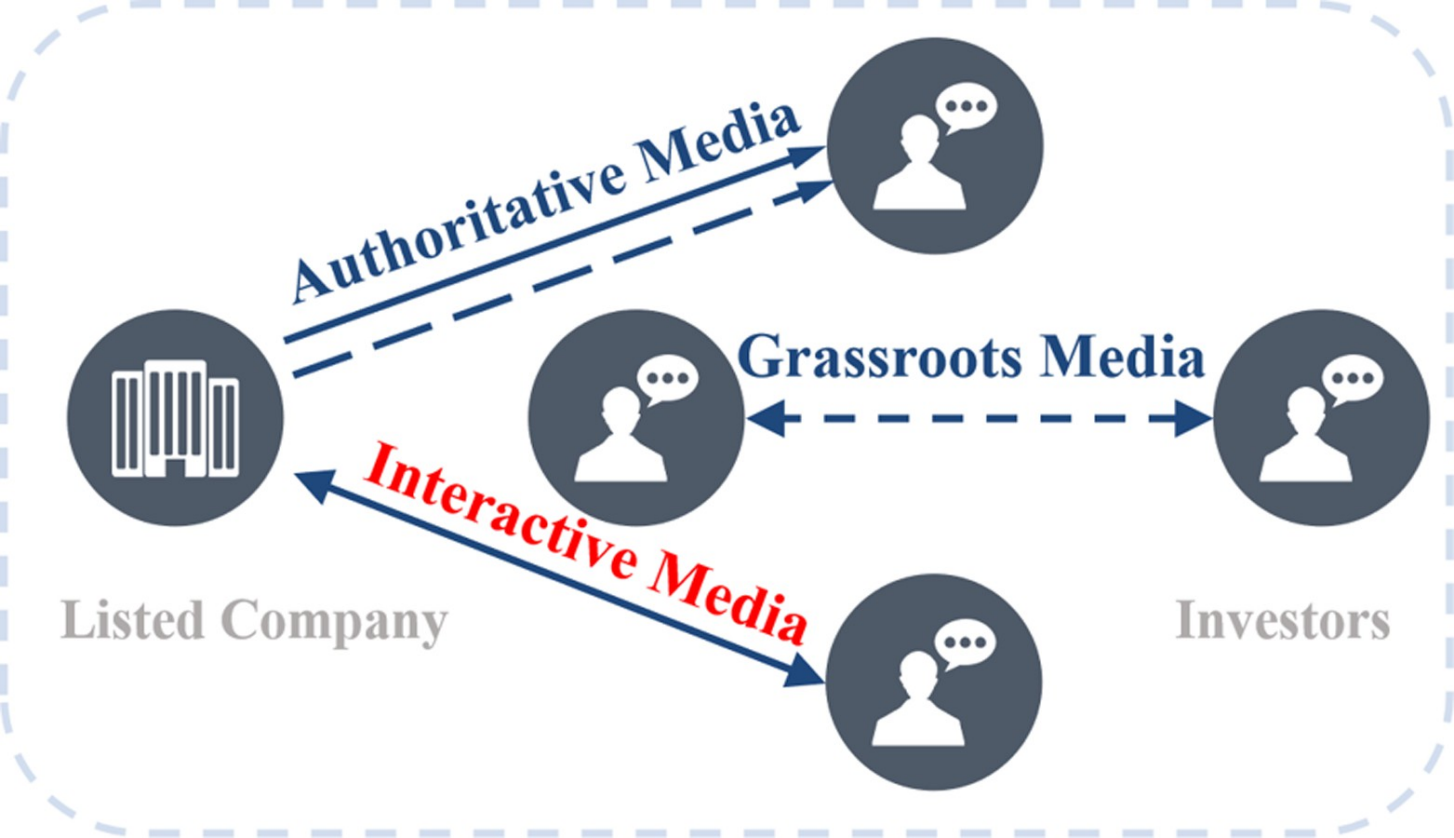
Media information flow diagram under different media development stages.

There are significant differences between emerging digital interactive platform and traditional official news website and investor social platform. First of all, the number of Q&A (interactive) texts in digital interactive media far exceeds the number of information releases on traditional media platforms [4]. According to the statistics of the Investor Education Center of Shenzhen Stock Exchange, in the first half of 2023, the number of interactive Q&A text has reached over 150000. The massive interactive information must contain valuable factors affecting stock price fluctuations, which is the research basis of this paper. Secondly, digital interactive media includes two main bodies, while the traditional official news website is only the unidirectional output of authoritative media to investors, and investor social media is only the discussion and exchange between investors. From this, it can be seen that digital interactive media has a bidirectional dual-agent characteristic. The mapping relationship needs to be converted into multiple correlation mapping. It needs to extract representative interaction factors (differences between two agency) and information factors (contents of each agency) to understand the specific influence mechanism in digital economy era. The information flow of media in different media development stages is shown in Fig 2.

Unfortunately, although digital interactive media has become main information release channel in Chinese stock market, research on digital interactive platform is still relatively few. Existing research mainly focuses on the statistical analysis of the number of questions related to listed companies, as a measure of investor attention. Few studies quantify emotions based on the content of Q&A texts. It is vital to reveal influente mechanism of digital interactive platform on stock performance. Digging out valuable interaction factors in depth would help to strengthen stock market transparency and protect investors’ interests. This paper uses web crawling technology to obtain a massive amount of interactive text from digital interactive platforms, extracts emotional and interactive factors of listed companies and investors through emotional quantification technology, and constructs a multiple linear regression model to reveal the influence mechanism of digital interactive media on the Chinese stock market in the digital economy era. The relevant robustness testing, endogeneity testing and heterogeneity testing support empirical conclusions.

This paper has at least three innovations:

First, this paper expands the scope of financial research data. This paper uses distributed multi-threaded crawler technology to obtain massive Q&A texts from public digital interaction platform, forming a comprehensive database of financial Q&A texts in the Chinese stock market, which is the foundation for studying the information interaction mechanism between listed companies and investors in the era of digital economy.

Secondly, this paper applies sentiment quantification to interactive text. This paper uses Sentiment Word Matching and sentiment quantification algorithm to quantify the sentiment of interactive information into more valuable and representative features, in order to understand the factors that truly significantly affect stock prices in digital interactive media.

Thirdly, in terms of the influence mechanism, considering the dual-agencies characteristics of digital interactive media, this paper incorporates the interaction factors between investors and listed companies into a Multiple Linear Regression Model to analyze the influence mechanism and operation mechanism of new digital media platforms on market stock prices from a new perspective.

## Literature review

### Different media development stages

The significant influence of media information on the financial market, especially the stock market, has been widely proven [5–8]. At different development stages of media, the influence on the financial market is very different. Scholars usually use different methods to study this impact from different perspectives. The information media has gone through three main stages: authoritative media, grassroots media, and interactive media. In authoritative media stage, most scholars mainly focused on information statistics such as newspaper headlines, financial news, financial statements, and company announcements to capture fluctuations in stock prices [8–11]. For example, Birz et al. [12] also demonstrated the significant shock of GDP-related news in the LexisNexis database on stock price volatility from 1991 to 2004.

The rapid development of the Internet has broken the limitations of news dissemination on social media. Investors can communicate directly through grassroots media. Grassroots media such as Twitter, Weibo, and Stock Bar have emerged and been popular. Textual discussion information from grassroots media becomes an indispensable factor in analyzing the dynamics of stock market [7, 13–18]. Antweiler and Frank [19] analyzed 1.5 million discussion messages on Yahoo Finance and demonstrated the correlation between investor communication and stock returns.

At the same time, the impact of investor sentiment extends beyond the stock market with the rapid development of online technologies. Studies such as Milas et al. [8] delve into the bond market, illustrating how changes in investor sentiment can influence Eurozone’s sovereign bond yields and spreads, reflecting the market’s risk perceptions. Dergiades et al. [7] elucidates the pivotal role of social media discourse and web search intensity in impacting European bonds markets, revealing that online interactions and queries specifically regarding the Greek debt crisis significantly inform short-term yield differentials in select GIIPS countries. In the realm of commodities, Gao and Süss [20] explores the relationship between news sentiment and commodity price movements, revealing that sentiment indicators can serve as early predictors of market trends.

**Fig 3:**
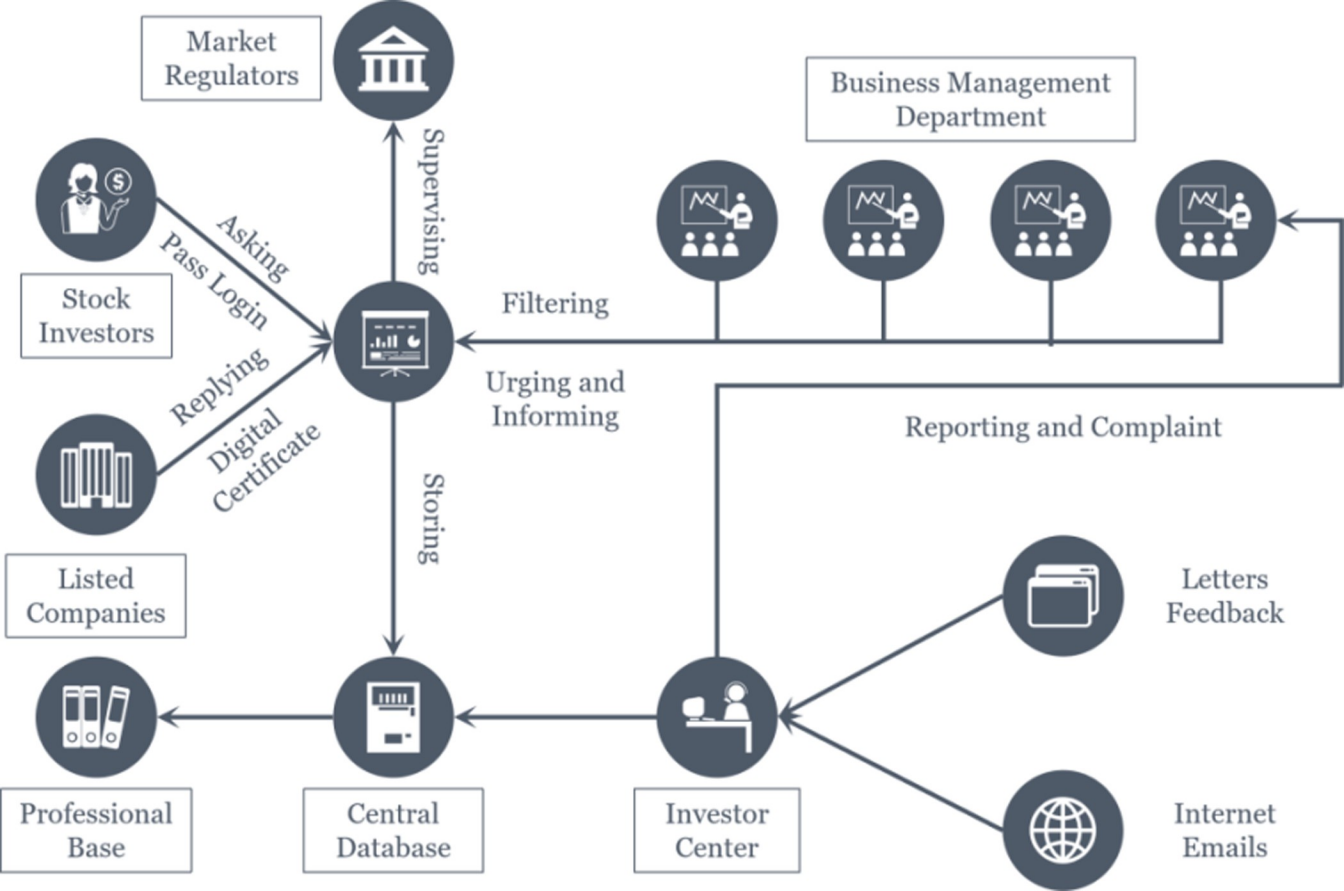
Panoramic network operation architecture diagram.

With the deep development of digital technology, digital interactive media has gradually become the main channel for listed companies information disclosure. The cloud architecture and big data technology in digital interactive media has facilitated direct communication between investors and listed companies. On digital interactive platforms, investors can ask questions to interested companies to understand their business status and development prospects. Listed companies can answer questions raised by investors and disclose information. Fig 3 shows the operational architecture of Panorama Network, a popular digital interactive platform in the Chinese market. It can be seen that investors log in to their online accounts and ask questions about listed companies. The business department of the listed company provides direct responses to questions related to the company. The emerging digital interactive media has sparked some Chinese scholars to explore its influence [21–24].

### Text information feature extraction

In the early stage of media development, official news website controlled the authority of information release. During this period, scholars mainly studied influence of news information on stock market through proportion of emotional words contained in "authoritative" news. Tetlock shows that the correlation between stock returns and proportion of emotional words is exceedingly significant, that is, if the news about listed company is highly optimistic, the company’s stock price will rise, vice versa [11]. Li et al. [25] used proportion of emotional words from the news to construct Public Sentiment Index (PSI), fused with basic information to predict stock price trend. Table 1 presents representative literature on stock market research under different media stages.

**Table 1:** Representative literature on stock market research under different media stages.

Media Stages	Literatures	Market Information			Analytical Method			Experimental Setting		
		Information Source	Information Scale	Target Market	Features/Factors	Empirical Models	Observed Indicators	Period	Evaluation Criteria	Frequency
Authoritative Media	Dergiades et al. (2015) [7]	Google Trends	98 Search Terms	DJIA	Change in Search Terms	Linear Regression	Stock Index	2004–2011	Stock Price Error	Week
	Gao and Süss (2015) [20]	Wiki	30 Wiki Articles	DJIA	Page Traffic	Linear Regression	Stock Index	2007.12.10–2012.04.30	Stock Price Error	Week
	Andersen et al. (2007) [10]	PRNewswire	500 Stocks	S&P500	News Content	k-Nearest Neighbor, Support Vector Machine	Stock Price	2002.04.01–2002.12.31	Trend Error	Minute
	Das and Chen (2007) [13]	Yahoo	127 Stocks	S&P500	Stock Price, News Content	Language Model	Stock Price	1999.10.15–2000.02.10	Trend Error	Hour
	Ding et al. (2004) [34]	Reuters, Bloomberg	15 Stocks	S&P500	News Events	Deep Neural Network, Support Vector Machine	Stock Index	2006.10.02–2013.11.21	Stock Price	Day
	Birz et al. (2011) [12]	LexisNexis	53 Stocks	S&P500	Market Data, News Features	Multiple Kernel Learning	Stock Price	2009.01.09–2014.01.09	Stock Price Trend	Day
Grassroots Media	Antweiler and Frank (2004) [19]	Yahoo Finance, Raging Bull	45 Stocks, 1.5 Million Texts	DJIA, XLK	Emotions, Number of Messages	Linear Regression	Revenue, Trading Volume	2000	Stock Price Error	Day
	Bollen et al.(2011) [14]	Twitter	9 Million Tweets	DJIA	Historical Stock Index, Sentiment	Self-organizing Fuzzy Neural Networks, Granger Causality	Stock Index	2008.02.28–2008.12.19	Trend Error	Day
	Li et al.(2014) [25]	Internet	89 Stocks	CSI100	Historical Stock Prices, News Content, Forum Sentiment	Support Vector Regression	Stock Price	2011.01.01–2011.12.31	Stock Price Error, Trend Error	Minute
	Yang et al. (2015) [16]	Twitter	12 Contracts	America	Twitter Sentiment, Quantity	Mutual Information	Contract for Difference	2012.11.12–2013.03.12	Stock Price	Hour
	Huang et al.(2016) [18]	Twitter	4 Major Stock Indices	America	Twitter Emotions	Convolutional Neural Network, Deep Neural Network	Stock Index	2009.06.12–2011.07.26	Stock Price Trend	Day
Interactive Media	Cen et al.(2016) [22]	Hudong Yi, Wind	1483 Stocks	SZSE	Investor Attention	Two-stage Least Squares	Return Rate, Stock Volatility, Liquidity	2010.02–2013.07	Robustness Test	Month
	Meng et al.(2020) [3]	Hudong Yi, CNRDS, Reset, CSMAR	Listed Companies on SZSE	SZSE	Negative Tone of Questions and Replies	Multiple Linear Regression(MLR)	Accumulated Abnormal Return	2010.01.01–2015.09.30	Robustness Test	Day
	Meng et al.(2019) [37]	Hudong Yi, Wind, Reset, CSMAR	All A-shares on SZSE	SZSE	Negative Tone of Questions and Replies	MLR	Risk of Stock Price Collapse	2010.01–2015.03	Instrumental Variable Method, Fixed Effects Model	Quarter
	Ding et al.(2018a) [2]	eHudong, CSMAR, Wind	946 A-shares on SSE	SSE	Unexpected Earnings, Interactivity, Institutional Investor Shareholding Ratio	MLR	Accumulated Abnormal Return Rate, Non Liquidity Indicator	2013.07.05–2015.11.30	Robustness Test	Quarter
	Ding et al.(2018b) [32]	eHudong, CSMAR	SSE A-shares with replies	SSE	Interactive Quality	MLR	Risk of Stock Price Collapse	2013.09–2015.11	Robustness Test	Month
	Li and Lu (2022) [33]	eHudong, CSMAR	1289 Listed Companies	SSE	Frequency and length of interactive communication	MLR	Stock Price Synchronicity	2014.01.01–2018.12.31	Cluster Robustness Standard Error, Endogeneity Test	Year

The development of network technology has led to a tremendous revolution in financial market. Investors can spread their opinions via social investment media such as stock bars or discussing forums. With the increase of media text content, the analysis of emotional dimensions has also changed from simple positive-negative judgments to high-dimensional Natural Language Processing (NLP) technology measurements. For example, Mitra and Mitra extracted eight dimensions of investor sentiment and discussed the impact of different dimensions of investor sentiment on stock performance [26]. With the increase of investor sentiment dimensions, research methods began to expand from the initial econometric model to the Machine Learning (ML) model to deeply capture influence of text information on stock price [27–29]. Dickinson and Hu used Deep Neural Networks (DNN) to find a strong correlation relationship between public sentiment in Twitter and stock prices [30]. For consumer-oriented companies such as Wal Mart and Microsoft, their stock prices are particularly affected by public sentiment. Huang et al. used Convolutional Neural Network (CNN) Algorithm to predict stock price trend from the perspective of public sentiment in Twitter, and found that CNN model performed better in most cases [18].

### Econometric multiple mapping model

How to build a correlation mapping model is the core of studying the media effect of the stock market. In the current research, most scholars use basic trading data (such as stock price, trading volume, turnover rate, return rate, etc.) and media information (such as text quantity, keywords, emotion, etc.) to build a correlation mapping model to capture influence factors on the stock fluctuations.

To analyze causal relationship between influence factors and stock fluctuations, econometric models are widely used to analyze the operating mechanism of the stock market. Among the models, the most classic model is Fama-French Three-factor Model [9]. This model takes the Market Value (MV), P/E ratio, and Book-to-market Ratio of listed companies as important indicators to explain the differences in stock returns.

In the field of information economics, many scholars have also used econometric models, considering text information factor and other factors as independent variables, return rate or price volatility as dependent variables [21, 22, 28]. For example, Huang et al. [18] controlled the list companies’ fundamental characteristics, collected the emotional tone in listed company’s press release and investors’ response, used Logistic Regression Model to estimate the Abnormal Positive Tone (ABTONE), which is positively correlated with current price, and negatively correlated with price delayed response of 1st and 2nd quarter. Karabulut [31] used Vector Autoregressive (VAR) Model to verify the predictability of National Happiness Index (GNH) on return rate and trading volume.

In the previous research of digital platforms, the number of questions [21, 22], the timeliness of replies [23], the clarity of replies [23], whether to open online interactive platforms [24], the number of text words [2, 32] are used as explanatory variables to verify causal relationship between attention degree from investors and stock price fluctuations. It can be seen that the previous research mainly relies on econometric models to study the correlation between media information and stock price. Digital interactive media contains a vast amount of text. Extracting factors solely from a statistical perspective may result in the loss of information value and affect the accuracy of analysis of the price trend.

In the digital economy era of information explosion, the research of digital interactive media cannot be limited to the previous research models. It is necessary to take into account the emotional characteristics contained in the massive text and the interactive characteristics based on the dual-agent characteristics, and build a diversified relationship mapping model, so as to truly reveal the impact mechanism. This paper firstly uses web crawling technology to obtain massive text information from the Panoramic Network. Then we use emotional word matching technology and emotional quantification algorithm to extract valuable influence factors. A Multiple Linear Regression (MLR) model was created to explore specific influence mechanism of digital interactive information on stock price performance.

## Experimental design

### Experimental framework

**Fig 4:**
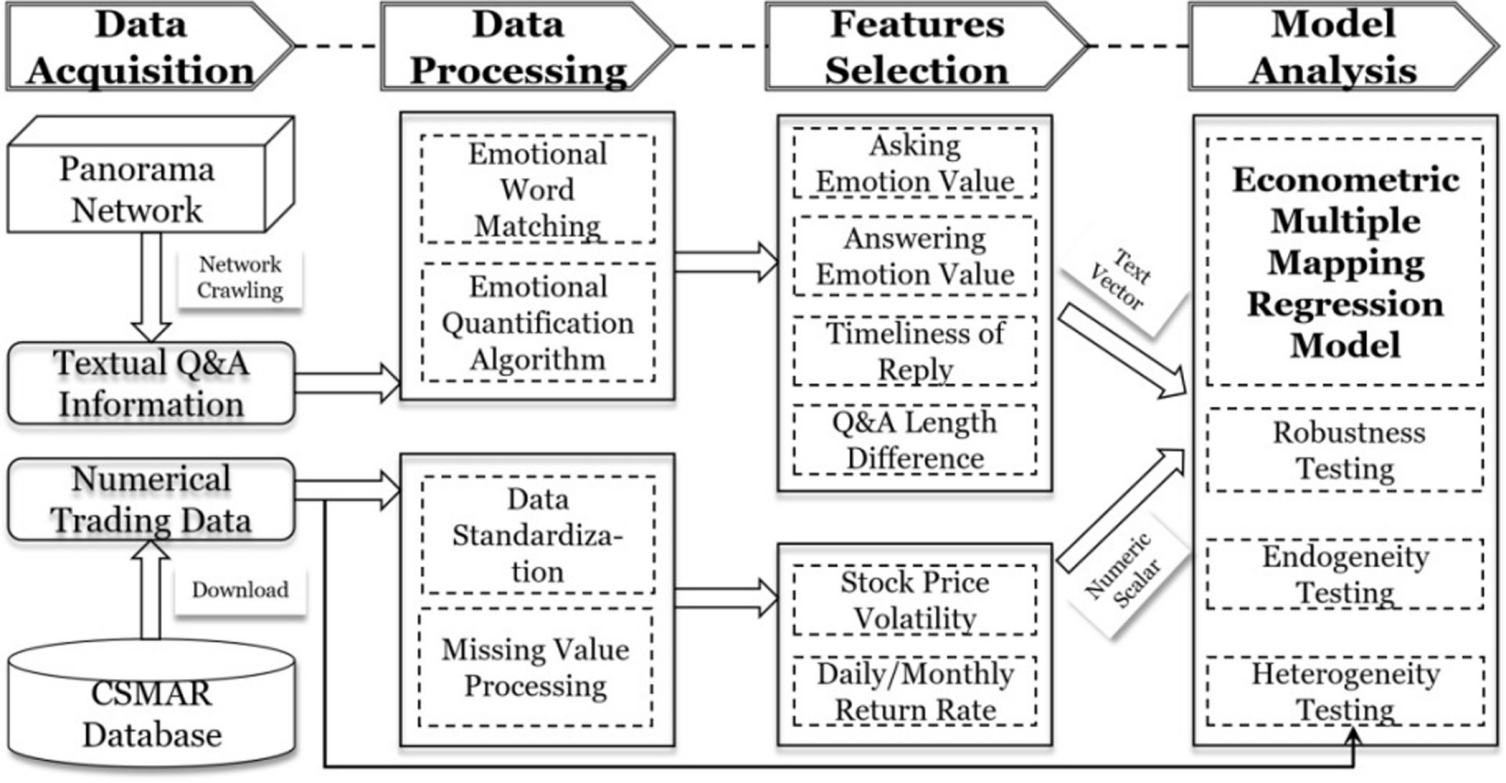
Research design and research process.

Digital interactive media has gradually become the main channel for direct communication between Chinese listed companies and investors, so it is necessary to explore the influence mechanism of digital interactive platforms. Fig 4 shows the research framework of this paper. In the data acquisition stage, on the one hand, this paper uses web crawler technology to capture text Q&A information from public digital interactive platform, and on the other hand, this paper downloads historical transaction data of listed companies from the CSMAR database for the corresponding period. In the data processing stage, after filtering the Q&A text information based on some criteria such as content length, emotional word matching technology is used to extract the number of positive/negative words in each question/answer text. And emotional quantification algorithm is used to calculate the emotional value of each question/answer text. In the feature selection stage, referring to previous research, influence factors and interaction factors such as the emotional value of investor questions, the emotional value of responses from listed companies, the timeliness of responses, the difference in word count between questions and answers are selected as independent variables, and the return rate as the dependent variable. In the model setting stage, a Multiple Linear Regression model is constructed to analyze the impact mechanism of Q&A information in digital interactive media on the operation of the stock market. Finally, this paper conducts a series of tests such as endogeneity test, robustness test, and heterogeneity test.

### Experimental data

For textual Q&A data, this paper uses an automatic crawler program to capture investors’ questions and listed companies’ responses from the Panoramic Network Interactive Platform, in order to extract specific influence factors and interactive factors. Panorama Network was established in 1999 and the interactive platform was opened to investors in 2014. It is now one of the largest investor interactive platforms in China. On this platform, this paper collected 62697 Q&A pairs from January 1, 2015 to December 31, 2021, totaling 117935 text messages. This paper filters the original text information according to the following criteria: deleting Q&A pairs without replies, deleting questions and replies with fewer than 10 words in length, and deleting Q&A pairs if the listed company involved misses historical transaction data and records. In the end, this paper obtained 54328 Q&A pairs, with a total of 108656 text information. The Q&A text involves 1970 listed companies on the Shanghai Stock Exchange, accounting for 71.14% of the total A-shares on the Shanghai Stock Exchange.

As for the numerical trading data, this paper downloads the relevant indicators of 1970 listed companies from the CSMAR database (one of the largest economic and financial research databases in China) from 2015 to 2021, such as daily returns, turnover, market value, and debt asset ratio.

### Emotional quantification

Digital interactive platform contains a massive amount of Q&A interactive text. The questions raised by investors have certain emotional characteristics (positive/negative). Investors may be influenced by rumors or real news, so they seek confirmation from listed companies on the platform. The response of listed companies to investors is to reduce the negative impact of rumors and disclose their operating conditions. In previous studies, scholars mainly measured investors’ attention to listed companies by counting the number of questions, which to some extent resulted in a huge loss of information value. This paper uses Emotion Word Matching method to count the positive and negative words in each question and answer, separating the positive and negative components in the Q&A pairs. Then the paper uses Emotion Quantification Algorithm to calculate the emotional value of each question and answer, in order to deeply analyze the emotional characteristics contained in the Q&A information.

**Fig 5:**
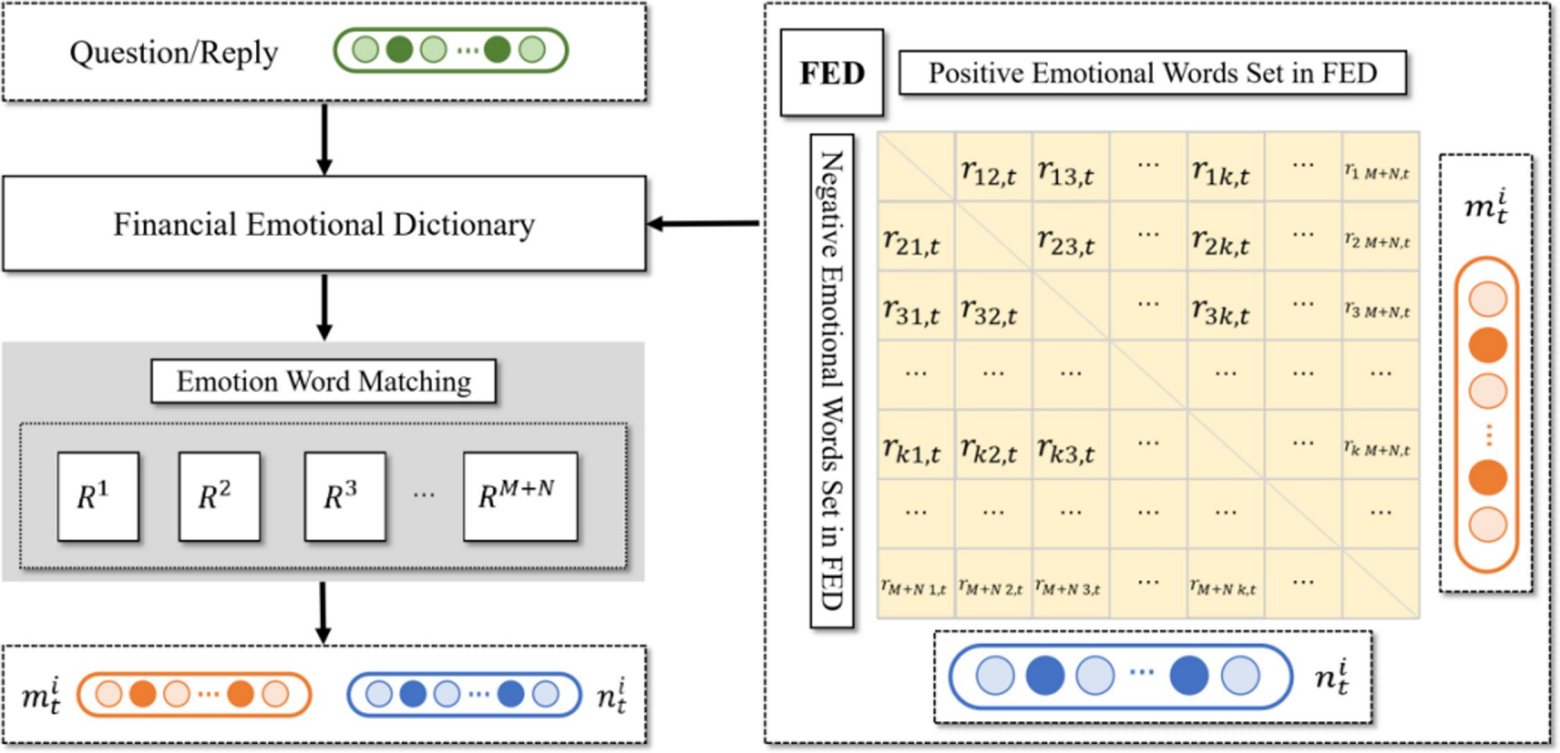
Emotional word matching flowchart.

Due to the fact that some important terms in the field of finance may change their emotional characteristics in the general field (such as "bull market"), this paper uses a professional Financial Emotional Dictionary (https://fife.swufe.edu.cn/sysgk/sysjj.htm) to capture emotional characteristics more accurately. After obtaining the Q&A text from panoramic network, this paper uses Emotional Word Matching to conduct emotional word statistics on the content of the text. Specifically, if each question/response contains a positive word from the Financial Emotional Dictionary, the number of positive words in that question/response will increase by 1. If each question/response contains a negative word from the Financial Emotional Dictionary, then the number of negative words in that question/response will increase by 1. The above operations can be automated through JAVA programming. Fig 5 is the flowchart of Emotional Word Matching.

When calculating text emotional values, this paper refers to the algorithm proposed by [29]. The specific calculation steps are as follows: after obtaining the number of positive/negative emotion words in each question/answer, calculate the mean and standard deviation of the number of positive/negative emotion words in the same listed company’s question/response, and standardize the number of positive/negative emotion words in each question/answer text. Finally, subtract the standardized number of positive emotional words from each question/answer text to obtain the emotional value of each question/answer text. The basic formula is as follows (1),

$$S_{ij}={\bar{P}}_{ij}-{\bar{N}}_{ij}=\frac{P_{ij}-\mu_{iP}}{\sigma_{iP}}-\frac{N_{ij}-\mu_{iN}}{\sigma_{iN}}$$ (1)

*S*_*ij*_ is the emotional value of the jth question/response of the i-th listed company; *P*_*ij*_ is the number of positive emotional words in the jth question/response of the i-th listed company; *N*_*ij*_ is the number of negative emotional words in the jth question/response of the i-th listed company; *μ_iP_* is the average number of positive emotional words in the questions/responses of the i-th listed company; *σ_iP_* is the standard deviation of the number of positive emotional words in the question/response of the i-th listed company; *μ_iN_* is the average number of negative emotional words in the questions/responses of the i-th listed company; *σiN* is the standard deviation of the number of negative emotional words in the question/response of the i-th listed company.

### Features selection

In previous research, most scholars mainly explored the correlation between the amount of external information and fluctuations of the stock market based on the information counting method [11, 25]. However, the massive text content on the digital interactive platform is very complex. Only measuring the degree of concern from the perspective of quantity can not fully reveal the impact mechanism. Some valuable information factors would be lost.

This paper attempts to analyze the massive text of digital interactive media from multiple dimensions. Firstly, the emotions of investors’ questions and listed companies’ responses are very important. On the one hand, investors may be influenced by real or false information in the market, and the relationship between emotions of questions and stock returns can reflect whether they will be "manipulated" by market information. On the other hand, responding to investors’ emotions by listed companies may alter their judgment in the process of verifying market information. Therefore, the emotional value of each investor problem and the emotional value of each response obtained through emotional quantification algorithm would become important influence factors.

Secondly, the timeliness of responses from listed companies is also an important indicator [21, 22]. Specifically, if a listed company can respond to investors’ questions in a timely manner, investors would not only be able to understand the company’s operating status and development prospects, but also consider the company’s management level to be high and its governance efficient. If a listed company fails to respond to investors in a timely manner, investors may overlook the questions raised or believe that the company does not have the confidence to respond positively, thereby affecting stock price performance. For this purpose, this paper refers to [33], constructing a timeliness indicator for responses.

Thirdly, the length of the content replied by listed companies reflects to some extent their attitude towards investors. If the questions raised by investors are long and include several sub questions, but the response from the listed company is short, it would be considered perfunctory and cause dissatisfaction among investors; On the contrary, if a listed company responds with a lot of content and elaborates on the concerns of investors, it would be considered as a high level of management. Referring to [23, 34], this paper calculates the word count of each question and response. And the attitude of listed companies could be measured by using the difference between the word count of each question and answer, which is also one of the interactive factors.

## Research hypothesis

The direct interaction on the Panorama not only optimizes investors’ information processing, but also has a positive influence on the management level of listed companies. Pinto and Asnani show that effectively eliminating the information acquisition behavior that interferes with information can optimize the information acquisition results [35].

The emotion, attitude and tone of the response of listed companies can be obtained by investors. It can help investors to form a price expectation close to the basic value. These micro processes can significantly affect the fluctuation of stock prices. The more investors’ price expectations tend to the basic value, the smaller the risk of stock price fluctuations [32, 33]. Relying on functional characteristics and institutional arrangements, the interaction of Panorama Network can enable investors to directly obtain replies from interested listed companies and make effective investment judgments. Therefore, the following assumption is proposed in this paper.

**H1:** The more positive, timely and conscientious the listed companies respond to investors’ questions, the more stable the stock price would be and the better their performance would be.

Investors ask questions on the digital interactive platform. According to the herd effect of behavioral finance, other investors will also be affected, thus amplifying the impact caused by investors’ questions [36]. In this view, this paper constructs the corresponding emotional factors of investors’ questions to reveal the impact of digital interactive media questions using emotional quantification. So this paper puts forward the following assumption.

**H2**: The more positive the emotion investors ask is, the better the stock price will perform.

### Data integration

This paper downloaded historical transaction data of 1970 listed companies from January 1, 2015 to December 31, 2021 from the CSMAR database, including daily returns, turnover rates, and other financial indicators. These numerical indicators, along with the influence factors extracted from textual information, are added to the Multiple Linear Regression Model. So it is necessary to match the features extracted from textual information with the numerical financial indicators according to specific rules. The specific rules are as follows, taking the matching of daily returns of listed companies and Q&A pairs as an example.

**Fig 6:**
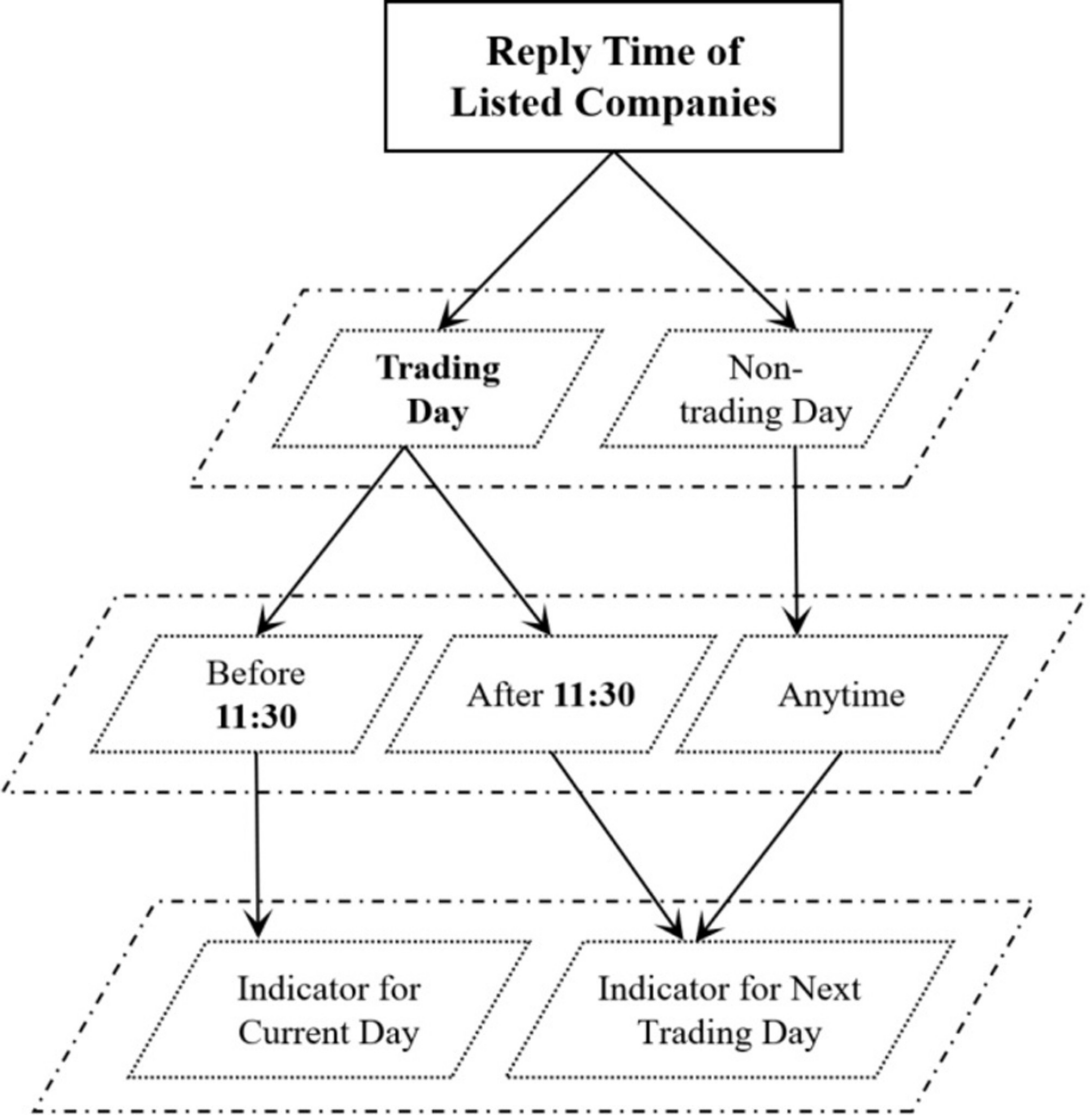
Rules for matching numerical financial indicators with textual quantitative indicators.

Generally speaking, investors would make investment decisions after receiving a response from listed companies, so this paper uses the response time of the listed company as a reference for matching return rate. Due to the time lag between a listed company responding to investors and investors making decisions based on their feedback, it is necessary to classify and match return rates here. If the listed company responds to investors before 11:30 (closing time), then match the Q&A pair with the daily return of the company on that trading day. If the listed company responds to investors after 11:30 (closing time), then match the Q&A pair with the daily return of the company on the next trading day. If a listed company replies to investors on a non-trading day, match the Q&A pair with the daily return rate of the company on the next trading day. Fig 6 shows the rules for matching numerical financial indicators with textual quantitative indicators. Besides, annual financial indicators are based on the year when the listed company responds to investors.

## Empirical analysis

### Model setting

This paper attempts to construct a Multiple Linear Regression Model to analyze the factors that truly affect stock price performance in digital interactive media texts and their and influence mechanisms. The explanatory variables of the basic regression model include: the emotional value of the question, the emotional value of the response, the timeliness of the response, the difference in the length of the question and answer, the daily turnover rate, and the interaction term of the emotional values of the question and answer. The dependent variable is the daily return rate of the listed company. The regression equation is shown in (2).


Ri,j=β0+β1×Replyi,j+β2×Quesi,j+β3×Lengthi,j+β4×Timei,j+β5×Turni,j+β6×Quesi,j⋅Replyi,j+εi,j (2)

The abbreviations and descriptions of model variables are shown in Table 2. ‘i’ represents the i-th listed company. ‘j’ represents the jth Q&A pair for the i-th listed company.

**Table 2:** Abbreviations and descriptions of variables in the regression model.

Variable Type	Symbol	Variable Descriptions
**Explained **V**ariable**	*R*	*R*_*ij*_ represents the daily return rate that matches the j-th Q&A pair of the i-th listed company. The specific matching rules have been detailed in the previous paragraph.
**Explanatory **V**ariable**	*Reply*	*Reply*_*ij*_ represents the emotional value of the response in the jth Q&A pair of the i-th listed company.
	*Ques*	*Ques*_*ij*_ represents the emotional value of the question in the jth Q&A pair of the i-th listed company.
	*Length*	*Length*_*ij*_ represents the difference between the length of the question and response in the jth Q&A pair from the i-th listed company. The calculation method is to subtract the number of words in the response from the number of words in the question.
	*Time*	*Time*_*ij*_ measures the timeliness of the reply in the jth Q&A pair from the i-th listed company. Referring to the approach of [33], this variable is set as a dummy variable. If a listed company responds to investor questions within five days, the value is 0. If a listed company responds in more than five days after an investor’s question is raised, the value is 1.
	*Turn*	*Turn*_*ij*_ represents the daily turnover rate that matches the jth Q&A pair of the i-th listed company.
	Ques* *Reply*	*Ques*_*ij*_ · *Reply*_*ij*_ represents the interaction term of the emotional values of the question and answer of the jth Q&A pair of the i-th listed company.
Control Variable [2, 3, 24, 37]	*ALR*	*ALR*_*ij*_ represents the asset liability ratio that matches the jth Q&A pair of the i-th listed company.
	*ROA*	*ROA*_*ij*_ represents the return on assets that matches the jth Q&A pair of the i-th listed company.
	*ROE*	*ROE*_*ij*_ represents the return on equity that matches the jth Q&A pair of the i-th listed company.
	*EPS*	*EPS*_*ij*_ represents the matching earnings per share for the jth Q&A pair of the i-th listed company.
	*DMV*	*DMV*_*ij*_ represents the daily market value that matches the j-th Q&A pair of the i-th listed company.
	*YMV*	*YMV*_*ij*_ represents the annual market value that matches the jth Q&A pair of the i-th listed company.
	*IND*	*IND*_*i*_ represents the industry to which the i-th listed company belongs.
	*AREA*	*AREA*_*i*_ represents the region where the i-th listed company belongs. This variable is set as a dummy variable, divided into the eastern region, central region, western region, and northeastern region.
	*YEAR*	*Year*_*i*_ represents the year of registration of the i-th listed company.

### Descriptive statistics

This paper conducted descriptive statistical analysis and Table 3 shows the results. The average daily return rate of the sample set of listed companies is 0.0735%, with a maximum of 29.35% (the increase limit for new stock issuance in the Chinese stock market is 44%) and a minimum of -11.21%. The data is consistent with the actual operation of the stock market and indicates that there is a huge difference in the return of Chinese listed companies. The average emotional value of replies from listed companies is 0.000064, with a maximum value of 41.72 and a minimum value of -13.71. This indicates that the tone of responses from listed companies tends to be positive, which is related to maintaining corporate images and relationships with investors. The average emotional value of question is 0.000582, with a maximum value of 16.88 and a minimum value of -12.48. The data is consistent with the complex psychology of the investors in the current stock market. The average value of Length is 45.76, ranging from -208 to 2076, indicating that listed companies tend to elaborate more on the situation and disclose information as much as possible. The average Time is 0.3374, indicating that most listed companies respond to investors’ questions within 5 days. The average value of Turn is 0.527%, and the maximum value is 1.527%, which indicates that the stock market is not active in the post financial crisis period and the COVID-19 epidemic period.

**Table 3:** Descriptive statistics of model variables.

	(1)	(2)	(3)	(4)	(5)
VARIABLES	N	mean	sd	min	max
R	54328	0.000735	0.029647	-0.112104	0.293459
Reply	54328	0.000064	2.272465	-13.71429	41.71651
Ques	54328	0.000582	1.767153	-12.48772	16.87941
Length	54328	45.765	93.421	-208	2076
Time	54328	0.3374	0.4732	0	1
Turn	54328	0.00527	0.00204	0.00280	0.01527
ALR	54328	0.456	0.333	0.008	5.681
ROA	54328	0.0115	0.2681	-7.7005	4.7079
ROE	54328	0.0299	0.4224	-4.5692	11.0403
EPS	54328	0.362	1.215	-7.731	35.000
DMV	54328	0.00870	0.04272	0.00036	1
YMV	54328	0.00760	0.04524	0.00031	1

### Empirical results

This paper constructs a Multiple Linear Regression Model, attempting to reveal the factors that can truly influence stock prices in the textual information of digital interactive media. This paper uses a fixed effects model based on the results of the Hausman Test. The regression results in the following text are all regression coefficients under panel fixed effects. Table 4 shows the outputs of the Simple Linear Regression Models and the Multiple Linear Regression Model.

**Table 4:** Outputs of the simple linear regression models and the multiple linear regression model.

	(1)	(2)	(3)	(4)	(5)	(6)	(7)
VARIABLES	R	R	R	R	R	R	R
Reply	0.0001431**	0.0000816	—	—	—	—	—
	(2.28)	(1.47)	—	—	—	—	—
Ques	0.0000595	—	0.0001286*	—	—	—	—
	(0.80)	—	(1.80)	—	—	—	—
Length	-4.27e-06***	—	—	-3e-06**	—	—	—
	(-2.84)	—	—	(-2.23)	—	—	—
Time	-0.0004046	—	—	—	-0.0003418	—	—
	(-1.51)	—	—	—	(-1.28)	—	—
Turn	0.721542***	—	—	—	—	0.4979***	—
	(11.63)	—	—	—	—	(9.07)	—
Ques* Reply	-0.0000243	—	—	—	—	—	-0.0000274
	(-0.90)	—	—	—	—	—	(-1.02)
Constant	-0.003***	0.001***	0.001***	0.001***	0.001***	0.001***	0.001***
	(-7.38)	(5.81)	(5.81)	(6.20)	(5.47)	(8.77)	(5.90)
Observations	54328	54328	54328	54328	54328	54328	54328
F test	0	0.143	0.0724	0.0255	0.201	0.0089	0.308
r2_a	0.00265	2.11e-05	4.10e-05	7.34e-05	1.16e-05	3.71e-05	7.20e-07
F	25.06	2.147	3.228	4.989	1.632	7.162	1.039

t-statistics in parentheses

*** p<0.01

** p<0.05

* p<0.1

In the Simple Linear Regression Models, the variables Length and Turn are significant at a significance level of 5%, while the other variables are not significant. In the Multiple Linear Regression Model, the regression coefficient of Reply is 0.0001431, indicating that for every unit increase in the emotional value of a listed company’s reply, on average, the daily return of the stock would increase by 0.01431%. This variable is significant at a significance level of 5%, indicating that investors are more concerned about the reply of listed companies and use it as a basis for decision-making. This is consistent with the Proximate Cause Effect Theoty in behavioral finance, where investors’ investment judgments depend on the latest responses received. From the perspective of listed companies, the positive tone of their response implies a high level of management and superior governance efficiency. The variable Ques is not significant, indicating that whether an investor raises positive or negative questions to a listed company, it does not represent their true investment attitude. Unlike the research hypothesis H1, the estimated coefficient of Length is negative and significant at the 1% significance level. This indicates that the fewer words a listed company responds, the better its stock price performance may be. A reasonable explanation is that when faced with questions with different emotions, listed companies would react differently. If the problem appears relatively negative, the listed company may provide a more detailed response, but this may be mistaken by investors as a "defense" and reduce their holdings of the stock. What investors need more is a streamlined and problem-solving response. The variable Time is not significant. Investors do not mind if listed companies do not respond in a timely manner. The estimated coefficient of turnover rate is 0.721542, which is significant at the 1% significance level. When the turnover rate is higher, stock trading becomes more active, and the stock price may slightly rise in the short term, which is consistent with the research conclusion of [36].

The adjusted R-square of the Multiple Regression Model is 0.00265, which is reasonable. Financial markets, characterized by their inherent complexity and the multitude of factors (an array of economic, political, psychological, and now digital factors) influencing stock price movements, often result in low R square in empirical finance research, especially when exploring new dimensions of market behavior such as the impact of digital interactive media (DIM). Research exploring the effects of investor sentiment, news, and other non-traditional factors on market returns frequently report lower R square [36, 38–40]. Kandel and Stambaugh [38] highlight the inherent challenges in predicting stock market movements, emphasizing the limited explanatory power of models. The regression of monthly stock returns on dividend yields produces an R-square equal to 0.0024. Tetlock et al. investigate the influence of news sentiment on stock market performance, demonstrating a modest explanatory power with adjusted R square values ranging from 0.001 to 0.002 [36].

The finding that investors’ questions about listed companies do not significantly affect stock price performance, while the responses from these companies do, invites a profound theoretical exploration within the framework of financial theories and information theory. The reasons why the responses from listed companies can significantly affect stock prices are as follows. This conclusion confirms the Framing Effect in behavioral finance. The Framing Effect explains how the presentation of information, including the tone and sentiment of corporate communications, can affect decision-making [41]. Thus, the impressions and attitudes in company responses may frame investor perceptions in a way that significantly impacts stock prices. Our observations also resonate with Signaling Theory, which suggests that companies communicate certain information to signal their quality or prospects to the market [42]. The qualitative aspects of these communications ‐ impressions and attitudes ‐ serve as signals that investors interpret as indicative of the company’s future performance or management’s confidence. The significant effect of company responses on stock prices can further be explained by the phenomena of information cascades and herd behavior. When influential companies issue replies loaded with positive impressions or attitudes, it can trigger an information cascade, where investors, irrespective of their private information, follow the actions of others based on the observed behavior. This herd behavior amplifies the impact of corporate communications on stock prices, as investors collectively interpret these qualitative cues as a consensus about the company’s prospects.

The analysis that investor questioning could not significantly affect stock prices is as follows. The EMH posits that stock prices reflect all available information [43]. Under this hypothesis, investor questions (public queries) do not introduce new, actionable information to the market and thus have a minimal impact on stock prices. Conversely, responses from companies can provide new, material information or clarify uncertainties, which the market then quickly assimilates, affecting stock prices. The concept of information asymmetry suggests that different market participants possess varying degrees of information about a company’s prospects [44]. Investor questions typically emerge from this asymmetry, representing attempts to reduce the informational gap between investors and company management. However, without the company’s acknowledgment or response, these questions alone do not resolve the asymmetry or significantly impact stock price performance.

The differential impact of investor questions and company responses on stock price performance is multifaceted, rooted in the dynamics of information dissemination, market psychology, and the theoretical underpinnings of financial markets. While investor queries highlight areas of interest or concern, it is the companies’ responses that carry the weight of new information, capable of altering market perceptions and stock valuations. This distinction underlines the critical role of corporate communication in financial markets, where the clarity, quality, and timeliness of information disclosure can significantly influence stock price trends.

### Endogeneity testing

The relationship between the direct interaction of digital interactive media and stock performance may also be endogenous in the relevant characteristics of the enterprise. For example, the reason why investors frequently interact with the listed companies through platforms such as "Panorama Network" is because the company has completed information disclosure and lots of external activities. These have attracted widespread attention of investors. These are all evidences of good operation of company. Therefore, there may exist endogeneity problem with the reciprocal causation between the dependent variable and the explanatory variables in the model. Compared to the Ordinary Least Squares Method (OLS), the advantage of Differences in Differences (DID) method is that it controls the reciprocal causation effect between the dependent variable and the independent variables [45]. Besides, Differences in Differences Model can also control the impact of unobservable individual heterogeneity on the dependent variable in panel dataset. Panorama Network began operating its online interactive platform in January 2014, providing a more effective exogenous impact scenario for adopting a Differences in Differences model. Therefore, in order to alleviate the endogeneity problem in the model and verify the causal relationship between digital interactive media interaction and stock price performance, this paper uses a Differences in Differences model to test.

The parallel trend test serves as a pivotal mechanism to validate the assumption that, in the absence of the treatment (i.e., the adoption of digital platforms), the treatment and control groups would have followed parallel paths over time. This assumption is fundamental to the DID methodology, as it underpins the credibility of attributing observed differences in outcomes to the treatment effect. In the parallel trend test, we use Parkinson’s volatility as the outcome indicator, and its calculation formula is as follows. The indicator attempts to capture the change of stock price within a day, which is more informative than the volatility based on the closing price [46].


$$\text{Parkinson}\;\text{Volatility}=\sqrt{\frac{1}{4\ln\left(2\right)}*\frac{1}{n}\sum_{i=1}^{n}{\left(\ln\left(\frac{H_{i}}{L_{i}}\right)\right)}^{2}}$$ (3)

**Fig 7:**
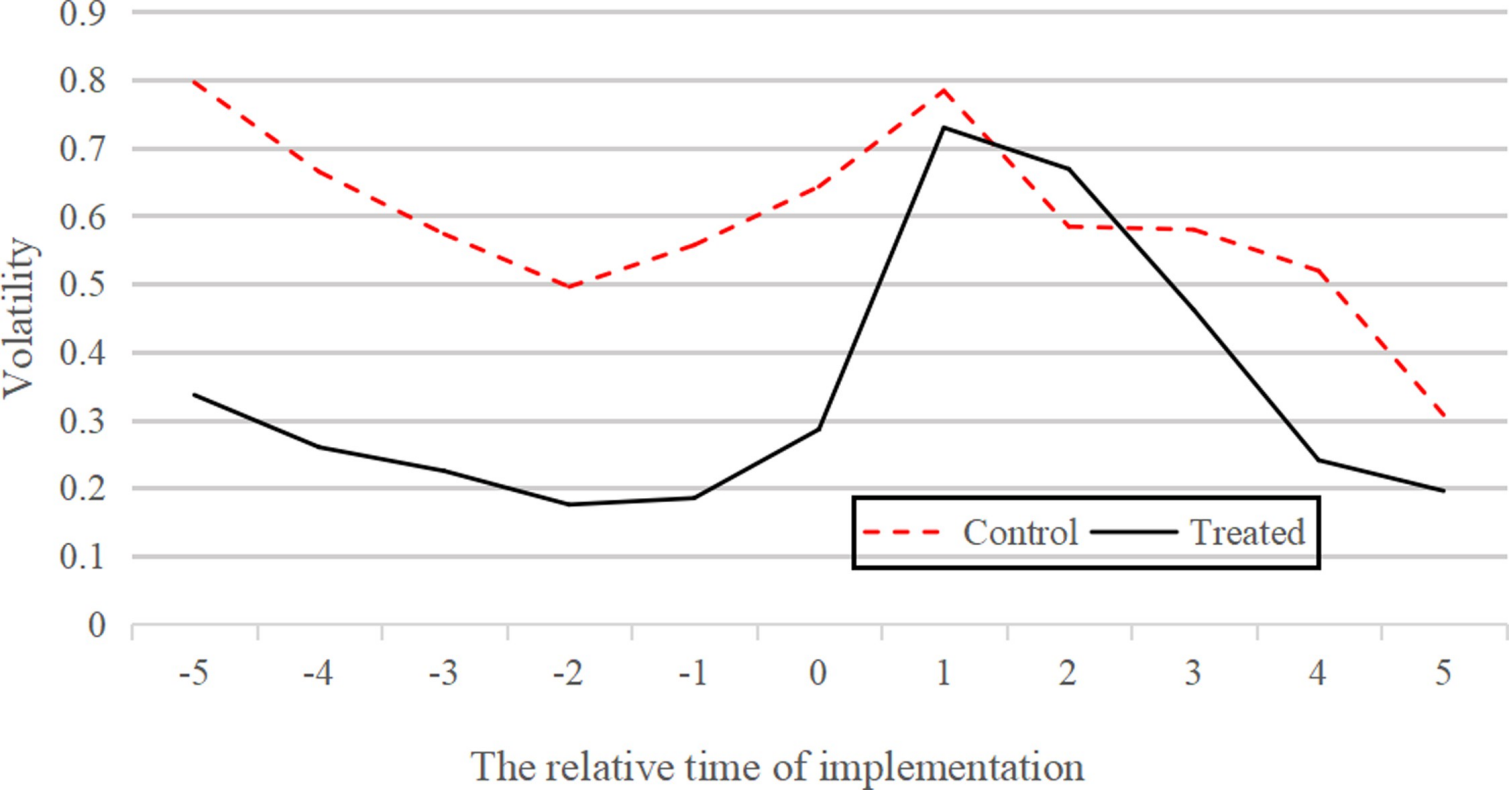
Parallel trend test.

In this paper, the monthly volatility of listed companies that have not joined the platform and that of listed companies that have joined the platform are averaged respectively, and the time window of 5 months before and after joining the platform is taken as the time window for parallel trend test. The time trend chart of parallel trend test is shown in Fig 7.

Prior to the intervention (at time 0), the volatility trends for both the treated and control companies exhibit a congruent trajectory, which corroborates the validity of parallel trend assumption. Post-intervention, a marked divergence is apparent, particularly at the zenith of the treated group’s volatility, thereby signaling the impact of the DIM. This divergence is a critical indicator of the treatment effect, reflecting that the observed changes in volatility are not merely artifacts of pre-existing trends, but rather a consequence of the joining digital interactive platform. Thus, the parallel trend assumption is satisfied.

This paper takes 2012–2015 as the sample period, and the corresponding Differences in Differences model is designed as follows:

$$\begin{array}{l} Fluc_{i,t}=\alpha_{0}+\alpha_{1}\times Treat_{i}+\alpha_{2}\times Post_{t}+\alpha_{3}\times Treat_{i}\cdot Post_{t}\\ +\alpha_{4}\times Turn_{i,t}+\alpha_{5}\times \ln\left(Volume_{i,t}\right)+\varepsilon_{i,t} \end{array}$$ (4)

This model uses daily sample data. ***Fluc*** represents the fluctuation range of a listed company’s stock price, which is calculated by subtracting the lowest price from the highest price on current trading day. *Fluc*_*i*,*t*_ represents the fluctuation range of stock price for the i-th listed company on day t. ***Treat*** represents the attribute of listed company. If a listed company joins a digital interactive platform, the value is 1, otherwise the value is 0. ***Post*** represents the time attribute, with a value of 0 before 2014 and a value of 1 after 2014. *Treat*_*i*_ ⋅ *Post*_*t*_ is the interaction term between whether a listed company joins an interactive platform and the time. *Turn*_*i*,*t*_ is the daily turnover rate of the i-th listed company on day t. *Volume*_*i*,*t*_ is the daily trading volume of the i-th listed company on day t. Due to the large variation in trading volume of different listed companies during different periods, logarithmic processing is conducted here in order to reduce volatility.

The regression results of the Differences in Differences Model are shown in Table 5. Columns (1) to (4) respectively show the influences of listed companies joining digital interactive platforms on their subsequent stock price fluctuations for one month, three months, six months, and one year. This paper mainly focuses on the the coefficient of interaction item Treat*Post *α*_3_. If the launch of digital interactive platform increase the stock price fluctuations, the regression coefficient would be significantly positive, and vice versa. The results show that after controlling for the fixed effects of the company, the coefficients of interaction item Treat*Post are -0.051, 0.051, 0.059, and 0.078. All are significant at the 1% level. This indicates that joining digital interactive media platforms can suppress stock price fluctuation in the short term, but it would increase stock price fluctuation in the medium-and long-term. Besides, the regression coefficient gradually increases, indicating that stock price fluctuations would gradually increase with the time the enterprise joins the platform.

**Table 5:** Regression results of the differences in differences model on daily data.

VARIABLES	(1)	(2)	(3)	(4)
	Fluc	Fluc	Fluc	Fluc
Treat	-0.589***	-0.668***	-0.762***	-0.317***
	(-5.66)	(-13.39)	(-10.55)	(-12.39)
Post	0.012	-0.096***	-0.132***	-0.206***
	(-1.23)	(-12.68)	(-15.24)	(-15.70)
Treat* Post	-0.051***	0.051***	0.059***	0.078***
	(-3.90)	(-4.29)	(-4.32)	(-4.15)
Turn	2.089***	3.059***	3.475***	4.718***
	(-5.93)	(-12.68)	(-15.42)	(-16.37)
Volume	0.199***	0.135***	0.117***	0.064***
	(-19.21)	(-19.63)	(-18.21)	(-8.77)
Constant	-2.419***	-1.511***	-1.228***	-0.422***
	(-16.13)	(-15.11)	(-13.13)	(-3.98)
Observations	42570	132256	257613	513559
Number of id	1077	1077	1077	1077
Company FE	YES	YES	YES	YES
r2_a	0.183	0.181	0.179	0.176
F	331.9	517.2	512.8	446.6

Robust t-statistics in parentheses

*** p<0.01

** p<0.05

* p<0.1

Due to the sensitivity of daily data, in order to further verify that the launch of digital interactive media significantly affects price fluctuations, this paper reconstructs a Differences in Differences Model using quarterly data. The formula is shown in (5),

$$\begin{array}{l} QFluc_{i,t}=\alpha_{0}+\alpha_{1}\times Treat_{i}+\alpha_{2}\times Post_{t}+\alpha_{3}\times Treat_{i}\cdot Post_{t}\\ +\alpha_{4}\times QTurn_{i,t}+\alpha_{5}\times \ln\left(QVolume_{i,t}\right)+\alpha_{6}\times ALR_{i,t}+\varepsilon_{i,t} \end{array}$$ (5)

***QFluc*** represents the average fluctuation range of a listed company’s stock price in a quarter, which is the arithmetic mean of the daily stock price fluctuation range in that quarter. *QFluc*_*i*,*t*_ represents the average fluctuation range of stock price for the i-th listed company in quarter t. If a listed company joins a digital interactive platform, the value of ***Treat*** is 1, otherwise the value is 0. ***Post*** still represents the time attribute, with a value of 0 before 2014 and a value of 1 after 2014. *Treat*_*i*_ · *Post*_*t*_ is the interaction term. *QTurn*_*i*,*t*_ is the arithmetic mean of daily turnover rate of the i-th listed company in quarter t. *QVolume*_*i*,*t*_ is the arithmetic mean of daily trading volume of the i-th listed company in quarter t. *ALR*_*i*,*t*_ represents the asset liability ratio of the i-th listed company in the t-quarter.

The regression results are shown in Table 6. Columns (1) to (4) respectively show the influences of listed companies joining digital interactive platforms on their quarterly stock price fluctuations for one quarter, two quarters, three quarters, and one year. The results show that the coefficients of interaction item Treat*Post are 0.020, 0.036, 0.032, and 0.075. All are significant at the 1% level. Similar to the previous regression results, the estimated coefficients of the intersection terms gradually increase over time. After a listed company joins the platform, the stock price fluctuation will increase with the joining time. The above results indicate that after the launch of the interactive platform on Panorama Network, the fluctuation range of stock prices of listed companies that joined the platform is significantly higher than that of listed companies that did not join the platform. This provides robust evidence for the significant impact of digital interactive media on corporate stock prices.

**Table 6:** Regression results of the differences in differences model on quarterly data.

	(1)	(2)	(3)	(4)
VARIABLES	QFluc	QFluc	QFluc	QFluc
Treat	-0.867***	-0.589***	-0.584***	-0.659***
	(-10.90)	(-13.01)	(-14.12)	(-5.35)
Post	-0.064***	-0.100***	-0.125***	-0.179***
	(-8.28)	(-12.31)	(-13.58)	(-14.66)
Treat* Post	0.020*	0.036***	0.032**	0.075***
	(1.91)	(2.87)	(2.20)	(4.19)
QTurn	4.118***	6.025***	6.691***	7.626***
	(5.10)	(10.17)	(11.79)	(13.38)
QVolumn	0.016	-0.023*	-0.064***	-0.090***
	(0.86)	(-1.75)	(-4.94)	(-6.38)
ALR	-0.157	-0.291***	-0.211**	-0.334***
	(-0.90)	(-2.76)	(-2.06)	(-3.16)
Constant	0.312	0.927***	1.514***	1.966***
	(1.16)	(4.61)	(7.52)	(8.96)
Observations	2154	4308	6462	8616
Number of id	1077	1077	1077	1077
Company FE	YES	YES	YES	YES
r2_a	0.195	0.190	0.185	0.225
F	51.84	97.39	107.9	145.0

Robust t-statistics in parentheses

*** p<0.01

** p<0.05

* p<0.1

Similar to the previous regression results, the estimated coefficients of the intersection terms gradually increase over time. After a listed company joins the platform, the stock price fluctuation would increase with the joining time. The above results indicate that after the launch of the interactive platform ‘Panorama Network’, the price fluctuation of listed companies that joined the platform is significantly larger than that of listed companies that did not join the platform. This provides robust evidence for the significant influence of digital interactive media on stock performances. In the long run, this is due to the fast information flow and frequent information interaction on digital interactive platforms. The time between the release of new information by listed companies and the receipt of information by investors has become shorter, allowing investors to make quick investment decisions. This increases the liquidity of stocks, thereby exacerbating stock price fluctuations.

### Robustness test

Some of the replies from listed companies to investors on digital interactive platforms lack substantive content. For example, "Thank you very much for your attention to our company and for your suggestions for its development". Most replies without substantive content have an emotional value close to 0. Overall, about 16000 non substantive responses were identified during the sample period. Although some short replies have been deleted during the data processing stage, considering that replies with no substantive content are invalid samples, this paper removes these Q&A pairs from the data. In addition, Chinese stock market may be systematically affected by the COVID-19 in 2020 and later. Referring to [47], this paper deletes the sample observations that occurred in 2020 and later.

The implementation of a Fixed Effect (FE) model within this paper acknowledges the unique characteristics of each entity. To encapsulate the dynamic impact of past price movements on returns, an autoregressive lag term is incorporated. This aligns with the inherent dynamism of financial markets [48]. This paper introduce ***Fluc*** as the autoregressive lag term. ***Fluc*** represents the fluctuation range of a listed company’s stock price, which is calculated by subtracting the lowest price from the highest price on current trading day. *Fluc*_*i*,*t*-1_ represents the fluctuation range of stock price for the i-th listed company on day t-1.

The regression results are shown in Table 7. It can be seen that the regression results are consistent with the basic regression results, with Reply being positive at the 5% significance level and Length being negative at the 5% significance level. After adding the autoregressive component related to stock price volatility, although the impact of sentiment value on stock price volatility is reduced, it is still significant at the level of 5%. This proves the robustness of the basis regression results.

**Table 7:** Robustness test results.

VARIABLES	(1)	CONTROLS	(1)
	R		
Reply	0.000094**	ALR	-0.0035***
	(2.05)		(-6.19)
Ques	0.000032	ROA	-0.0021**
	(0.75)		(-2.36)
Length	-5.88e-06**	ROE	0.0043***
	(-1.99)		(11.14)
Time	-0.000314	EPS	0.0000***
	(-0.45)		(2.75)
Turn	0.599328***	DMV	0.1421***
	(10.92)		(8.74)
Fluc	-0.291**	YMV	-0.166***
	(2.46)		(-9.03)
Constant	-0.00049**	IND	-0.00023**
	(-1.99)		(-2.52)
R-squared	0.0578	AREA	0.00039***
F test	0		(7.49)
r2_a	0.0576	YEAR	0.00017***
F	246.93		(3.67)

t-statistics in parentheses

*** p<0.01

** p<0.05

* p<0.1

### Heterogeneity testings

The previous testing results support the hypothesis that direct interaction on digital interactive platforms can influence stock price performance. However, the degree of influence may vary depending on the company’s operating conditions and attribute characteristics. Therefore, this paper further examines whether different characteristics of listed companies would bring about heterogeneous effects of digital interactive platforms on stock price performance from three aspects: region, capital size, and industry.

Given the complexities inherent in Chinese regional economic development and the significant disparities across different economic regions, utilizing the division of Chinese economic regions as a basis for heterogeneity testing is both theoretically and empirically sound. Firstly, the Efficient Market Hypothesis (EMH) suggests that prices reflect all available information [43]. However, in the context of Chinese diverse economic landscape, information asymmetry and regional economic policies can lead to differential market efficiencies. This variation provides a robust ground for analyzing the impact of regional economic characteristics on the degree of influence of digital interactive media. Secondly, different economic regions in China have unique risk profiles based on their GDP growth rates, unemployment rates, and levels of innovation [49]. According to Capital Asset Pricing Model (CAPM), these regional risk factors can significantly affect local stock market performance and interaction between investors and companies. Thirdly, regions experiencing positive economic growth or high levels of innovation may attract more securities analysts. Securities analysts process and sort out the original information released by listed companies, such as performance forecasts and annual financial reports, to extract valuable information and provide it to capital market investors [50]. This phenomenon will have a certain impact on the popularization of digital interactive media in various regions. Lastly, the theory of economic geography suggests that the spatial distribution of economic activities influences economic outcomes [51]. This theory supports the segmentation of China into different economic regions for financial analysis, as the geographic and economic heterogeneity can lead to varying interaction performances.

The division into economic regions is not merely a geographical distinction but a strategic analytical choice aimed at capturing the nuanced variations in market responses to digital interactive media (DIM) across different economic landscapes. China is currently divided into four major economic regions: the eastern region, the northeast region, the central region, and the western region. While market value and industry classifications provide critical layers of analysis, they may not fully account for regional economic policies, local investor sentiment, and specific regional economic cycles that can significantly affect market behavior. Based on above classification convention, this paper divides the regions of the listed companies into four categories for regression analysis. The regression results are shown in Table 8.

**Table 8:** Heterogeneity testing results of economic regions.

Variables	West	Central	East	Northeast
	R	R	R	R
Reply	0.0004897***	-0.0000102	0.0001205	-0.0001432
	(2.99)	(-0.06)	(1.59)	(-0.50)
Ques	-0.000144	0.0000562	0.0001017	0.000157
	(-0.72)	(0.25)	(1.15)	(0.47)
Length	-0.00000227	0.000000816	-0.00000522***	0.00000388
	(-0.54)	(0.18)	(-2.94)	(0.64)
Time	-0.0044229***	0.0046069***	0.00000651	-0.0055788***
	(-6.10)	(5.79)	(0.02)	(-4.71)
Turn	2.252091***	0.537522**	0.4096292***	2.398901***
	(13.25)	(2.56)	(5.70)	(7.42)
Ques* Reply	0.0000394	-0.0000094	-0.0000446	0.0000647
	(0.57)	(-0.11)	(-1.39)	(0.54)
Constant	-0.006***	-0.005***	-0.002***	-0.008***
	(-5.46)	(-4.15)	(-4.24)	(-4.41)
Observations	7304	6166	38649	2209
r2_a	0.0302	0.00575	0.00108	0.0313
F	38.90	6.939	7.956	12.88

t-statistics in parentheses

*** p<0.01

** p<0.05

* p<0.1

Only the Reply of listed companies in the western region significantly affects the return rate at a significance level of 1%. The western region is located inland and has relatively few information connections with the outside. The information disclosure of listed companies needs to be improved. Digital interactive media has become the main communication channel for investors to understand listed companies. Investors frequently communicate with listed companies in the western region in order to obtain more information. Only the Length of listed companies in the eastern region significantly shocks the return rate at a significance level of 1%. The eastern region has developed network technology and smooth information exchange. Once a listed company issues a response, it can be verified in the market. Long speeches are considered deliberate whitewashing to reduce stock holdings. Similarly, Time in the eastern region is not significant, while Time in other regions is significant. The information disclosure of listed companies in the eastern region is very comprehensive, and investors can learn about the relevant operational situation through various channels. Investors in other regions rely more on replies from digital interactive platforms as a basis for investment.

Referring to [33], this paper divides the circulating market value of listed companies into three categories: small-scale (below 100 million), medium-sized (between 100 million and 1 billion), and large-scale (over 1 billion). The expression results are shown in Table 9. Only medium-sized listed companies have a positive Reply and Length at a significance level of 5%. Due to the comprehensive governance system of large-scale enterprises, investors generally believe in and support large-scale enterprises. They have a skeptical attitude towards small-scale enterprises. So the replies of these two types of enterprises is unlikely to affect investors’ judgment. Time of large-scale enterprises is negative at a significance level of 1%. This indicates that investors have a certain mindset, believing that large-scale enterprises have more business and are unlikely to respond in a timely manner.

**Table 9:** Heterogeneity testing results of circulating market value.

VARIABLES	Large	Medium	Small
	R	R	R
Reply	0.0000912	0.0001693**	-0.0015692
	(0.78)	(2.30)	(-1.54)
Ques	0.0000229	0.0000714	-0.0003361
	(0.16)	(0.82)	(-0.39)
Length	-0.00000225	-0.00000551***	0.000354*
	(-0.81)	(-3.12)	(1.78)
Time	-0.003135***	0.0001732	0.0038396
	(-6.32)	(0.55)	(1.38)
Turn	1.413002***	0.5230292***	2.714977***
	(12.49)	(7.17)	(2.97)
Ques*Reply	0.0000179	-0.0000349	-0.0002456
	(0.36)	(-1.11)	(-0.41)
Constant	-0.003***	-0.002***	-0.014***
	(-4.85)	(-5.54)	(-2.88)
Observations	11313	42713	302
r2_a	0.0159	0.00144	0.0346
F	31.39	11.29	2.796

Referring to the "National Economic Industry Classification" implemented by the National Bureau of Statistics of China in 2017, this paper divides the sample of listed companies into 20 categories and performs regression analysis separately. Table 10 shows the testing results of five major industries.

**Table 10:** Heterogeneity testing results of industry.

VARIABLES	Manufacturing	Wholesale and Retail	Real Estate	Agriculture, Forestry, Animal Husbandry, Fishery	Accommodation Catering
	R	R	R	R	R
Reply	0.0000181	-0.0001248	0.000801**	-0.0004579	0.0012822
	(0.22)	(-0.46)	(2.28)	(-0.68)	(1.29)
Ques	0.0000416	0.0000262	-0.0003293	0.000886	0.001532
	(0.42)	(0.09)	(-0.71)	(1.14)	(1.15)
Length	0.00000124	-0.000023***	-0.000043***	0.0000511***	-0.0000413*
	(0.60)	(-3.33)	(-5.53)	(2.66)	(-1.76)
Time	-0.0002417	0.0012348	0.003991**	-0.0101909***	-0.0130082***
	(-0.69)	(1.13)	(2.30)	(-3.68)	(-3.66)
Turn	0.5541231***	0.3756954	1.81479***	2.85866***	9.723478***
	(6.84)	(1.50)	(5.15)	(4.15)	(5.81)
Ques*Reply	-0.0000331	0.0000341	-0.000049	0.000126	-0.000382
	(-0.94)	(0.28)	(-0.31)	(0.36)	(-0.68)
Constant	-0.002***	-0.003**	-0.019***	-0.009**	-0.034***
	(-4.05)	(-2.09)	(-8.63)	(-2.11)	(-4.51)
Observations	33086	2143	1255	424	44
R-squared	0.001	0.009	0.050	0.093	0.538
F test	7.72e-09	0.00555	0	3.28e-07	3.97e-05
r2_a	0.00130	0.00573	0.0452	0.0796	0.463
F	8.160	3.058	10.90	7.095	7.177

It can be seen that the Reply, Length, and Time of real estate enterprises are all significant at a significance level of 5%. The real estate industry is closely related to people’s livelihoods. It is also a barometer of the economy, which can reflect the operation of the economy. So investors ask real estate companies to obtain information about the macroeconomic performance. A streamlined, positive, and fast response can significantly improve the stock of real estate listed companies. The Length and Time of agricultural, forestry, animal husbandry, and fishing enterprises are significant at a significance level of 1%. Agriculture, forestry, animal husbandry, and fishery are the foundation of a country. So a detailed response can boost the confidence of investors in the stock market, thereby driving returns.

## Conclusion

Currently, digital interactive media has become the mainstream channel for investors to obtain information and listed companies to disclose information in the Chinese stock market. Digital interactive media contains a massive amount of text and has a dual-agency characteristic. This has significantly shocked the Chinese stock market and capital information environment, providing new challenges and opportunities for the mechanism research of stock market. The use of text analysis technology to explore the valuable factors in interactive content and explore the market function of the interaction ability between investors and listed companies is of great significance for improving the efficiency of stock market operation and enriching stock market research literature.

This paper firstly uses web crawling technology to obtain approximately 110000 Q&A text information from the Panoramic Network from 2015 to 2021. Then we use big data text analysis technology and emotional quantification algorithm to extract valuable influencing factors and interactive factors from the massive text. They are: the emotional values of the questions and the replies, the timeliness of the response, the difference in the length of the question and answer. A Multiple Linear Regression (MLR) model was created to explore specific influence mechanism of digital interactive information on stock price performance. The basic regression results indicate that, Reply is significant at a significance level of 5%. This is consistent with the Proximate Cause Effect Theoty in behavioral finance. The variable Ques is not significant. Unlike the research hypothesis H1, the estimated coefficient of Length is negative and significant at the 1% significance level. Investors need concise and positive responses.

In order to alleviate the endogeneity problem and verify the causal relationship between digital interactive media interaction and stock price performance, this paper uses a Differences in Differences model to test. The results provides robust evidence for the significant influence of digital interactive media on stock performances.

In the robustness test, this paper removes non substantive Q&A pairs from the data and deletes the sample observations that occurred in 2020 and later to regress again. The regression results are consistent with the basic regression results. Then this paper further examines whether different characteristics of listed companies would bring about heterogeneous effects of digital interactive platforms on stock price performance from three aspects: region, capital size, and industry. The results show that the response sentiment of Western, medium-sized, and real estate listed companies would significantly impact stock prices. The response length of eastern, medium-sized, real estate, and wholesale and retail listed companies would affect stock performance.

This paper has at least three innovations. This paper expands the scope of financial research data. Massive Q&A texts from public digital interaction platform form a comprehensive database of financial Q&A texts in the Chinese stock market. This paper uses Sentiment Word Matching and sentiment quantification algorithm to quantify the sentiment of interactive information into more valuable and representative features. Then, this paper incorporates the interaction factors between investors and listed companies into a Multiple Linear Regression Model to analyze the influence mechanism and operation mechanism of new digital media platforms on market stock prices from a new perspective.

## Future work

### Addressing the potential manipulation of DIM

This paper omitted the strategic ’operation’ of digital interactive media by publicly listed companies to disseminate misleading information. This oversight opens a fertile ground for future research: how might artificial intelligence technologies be harnessed to more adeptly discern the authenticity of corporate answers? A promising exploration would be the development of sophisticated AI algorithms capable of parsing nuanced corporate discourse and flagging potential disinformation. Such advancements would not only refine the veracity of media content analysis but also enhance the reliability of market sentiment indicators derived from these interactions. Future work could leverage more sophisticated NLP and Machine Learning techniques to better capture the nuances of investor sentiment, differentiate between genuine inquiries and noise, and identify valuable features and predictive signals within the vast volumes of digital interactions.

### Integration and analysis of multi-platform data

The second limitation concerns the scope of data sources. The current data of the paper is constrained by a singular DIM platform, although it is one of the leading DIM platforms in China. Future research should aim to cast a wider net by aggregating and scrutinizing Q&A texts from all leading Chinese digital media platforms. The challenge lies in the effective synthesis of these diverse data streams and the elimination of redundancies to fortify the empirical robustness of our findings. Achieving this would not only validate the conclusions drawn but also provide a more comprehensive understanding of the digital interactive media landscape’s impact on stock volatility.

### Panel data non-causality testing

This paper uses the Difference-in-Differences (DID) method to verify the causal relationship between the advent of digital interactive media platforms and the volatility of stock prices. Nevertheless, it is imperative to acknowledge the potential reciprocity of this relationship, particularly in instances of precipitous stock price declines. Such scenarios may catalyze significant shifts in investors’ questions, underscoring the importance of probing into the reverse causality. In light of the heterogeneous panel data presented in this paper, the non-causal panel data test proposed by [52] stands as a promising empirical approach for future examination. The exploration of this bidirectional causality is crucial for an intricate comprehension of the dynamics governing investor engagement on digital interactive media in response to stock market fluctuations.

### Interactive effects model among stocks

Acknowledging the intricate web of interactions among stocks catalyzed by investor-company communications on DIM platforms, future research could employ the interactive effects model by [53]. This model facilitates a deeper dive into how responses from companies influence not just the querying investor’s sentiments and decisions but also create a ripple effect impacting other stocks and the broader financial market. Such an analysis could unravel the interconnectedness of investor sentiments and market dynamics, highlighting the systemic implications of digital interactions.

## Supporting information

S1 Data (ZIP)
